# Environmental Bacteriophages of the Emerging Enterobacterial Phytopathogen, *Dickeya solani*, Show Genomic Conservation and Capacity for Horizontal Gene Transfer between Their Bacterial Hosts

**DOI:** 10.3389/fmicb.2017.01654

**Published:** 2017-08-30

**Authors:** Andrew Day, Jiyoon Ahn, Xinzhe Fang, George P. C. Salmond

**Affiliations:** Department of Biochemistry, University of Cambridge Cambridge, United Kingdom

**Keywords:** *Dickeya solani*, bacteriophage, environmental viruses, phytopathogen, horizontal gene transfer

## Abstract

*Dickeya solani* is an economically important phytopathogen widespread in mainland Europe that can reduce potato crop yields by 25%. There are no effective environmentally-acceptable chemical systems available for diseases caused by *Dickeya*. Bacteriophages have been suggested for use in biocontrol of this pathogen in the field, and limited field trials have been conducted. To date only a small number of bacteriophages capable of infecting *D. solani* have been isolated and characterized, and so there is a need to expand the repertoire of phages that may have potential utility in phage therapy strategies. Here we describe 67 bacteriophages from environmental sources, the majority of which are members of the viral family *Myoviridae*. Full genomic sequencing of two isolates revealed a high degree of DNA identity with *D. solani* bacteriophages isolated in Europe in the past 5 years, suggesting a wide ecological distribution of this phage family. Transduction experiments showed that the majority of the new environmental bacteriophages are capable of facilitating efficient horizontal gene transfer. The possible risk of unintentional transfer of virulence or antibiotic resistance genes between hosts susceptible to transducing phages cautions against their environmental use for biocontrol, until specific phages are fully tested for transduction capabilities.

## 1. Introduction

The enterobacterial genus, *Dickeya*, currently consists of six phytopathogenic species that can cause severe disease in economically important crops, including tomato, chicory, and potato (Reverchon and Nasser, 2013). The first report of *Dickeya* (previously known as *Erwinia chrysanthemi*) infecting European potatoes came from the Netherlands in the 1970s (Maas Geesteranus HP, 1972). Until 2004, almost all European potato isolates of *Dickeya* were assigned as *Dickeya dianthicola*, which has a broad host range across both nutritional and ornamental species (Toth et al., 2011). In the past 10 years however, three groups have independently identified a new clade of *Dickeya* in European potato isolates (Laurila et al., 2008; Parkinson et al., 2009; Sławiak et al., 2009). In 2014 this led to the classification of a new species; *Dickeya solani* (van der Wolf et al., 2014).

*Dickeya solani* is more aggressive than other *Dickeya* species, able to spread more easily through the plant vascular system and survive at higher temperatures than *D. dianthicola* (Toth et al., 2011). It is currently the predominant potato pathogen in Europe and in 2010 Scotland became the first country to introduce specific legislation aimed at preventing the establishment of *D. solani* in its seed industry (Mansfield et al., 2012).

In Israel a reduction in yield of up to 25% was observed in potatoes exposed to *Dickeya* species (Tsror et al., 2009). This imposes a significant economic cost, and consequently has led to research into methods for control of these virulent phytopathogens. In the absence of any effective chemical control systems, bacterial viruses (bacteriophages; phages) have been suggested as potential biocontrol tools and several studies have isolated phages capable of infecting *Dickeya* species (Adriaenssens et al., 2012b; Czajkowski et al., 2014, 2015; Matilla et al., 2014). Their potential use as biocontrol agents has been trialed both in the lab and in the field (Adriaenssens et al., 2012b) and these studies showed a “therapeutic” outcome with an increase in yield of the potato crop. Because of the potential utility of specific phages as therapeutic agents in potato soft rot control experiments, there is value in investigating a wider range of *Dickeya* phages. However, prior work has shown that a previously isolated *D. solani* phage is capable of generalized transduction of both chromosomal and plasmid markers (Matilla et al., 2014). The European Medicines Agency, among others, has stated that it is “important to ensure that therapeutic phages do not carry out generalized transduction” (Pelfrene et al., 2016), and therefore this is an important consideration as some *Dickeya* phages may not have been fully tested for generalized transduction capacity before field trials. This study therefore aimed to isolate and characterize a larger repertoire of new environmental phages against *D. solani* and investigate their potential for generalized transduction.

## 2. Results

### 2.1. Isolation and classification

**Figure 1:**
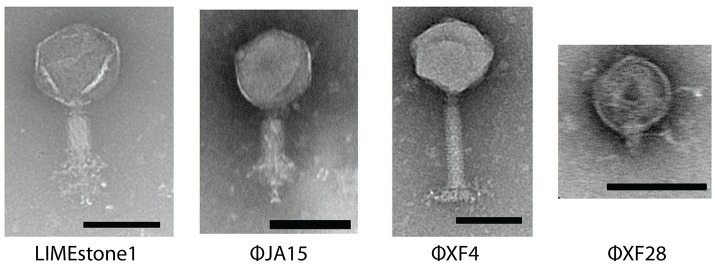
Transmission electron micrographs of four *Dickeya* phages. LIMEstone1 is a previously characterized *Dickeya* phage (Adriaenssens et al., 2012b). ϕJA15 and ϕXF4 are phages of the *Myoviridae* family exhibiting tail spikes as opposed to tail fibers. ϕXF28 is a phage of the *Podoviridae* family. Scale bars represent 100 nm.

Sixty-seven phages were isolated using standard enrichment techniques from both treated sewage effluent and river water between 2013 and 2015 using *D. solani* MK10 as the host organism. Transmission electron microscopy (TEM) showed two different morphological groups, a selection of which are shown in Figure 1 alongside the previously characterized phage LIMEstone1 (Adriaenssens et al., 2012b).

Of 24 phages imaged, all possessed an icosahedral head and a tail, placing them in the order *Caudovirales*. Three possessed short tails, characteristic of the family *Podoviridae* (such as ϕXF28 in the last panel of Figure 1) whilst the rest possessed longer contractile tails and belong to the family *Myoviridae*. The 21 *Myoviridae* members did not appear to possess the tail fibers characteristic of the family. Instead, short tail spikes were observed, (first three panels of Figure 1), and these are generally associated with the family *Podoviridae*.

### 2.2. Transduction

**Figure 2:**
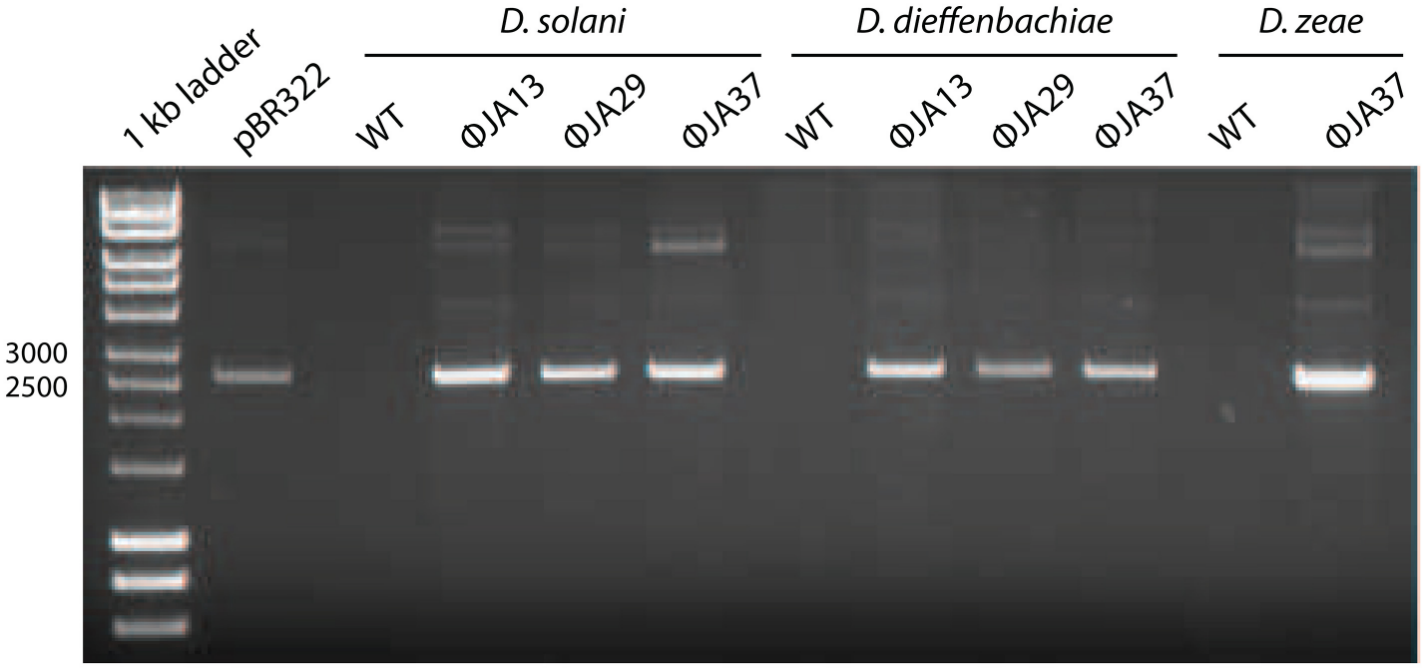
pBR322 was first used to transform *Dickeya solani* MK10 before the creation of lysates of ϕJA13, ϕJA29, or ϕJA37 on the recombinant host. These lysates were then used to infect *Dickeya solani, D. dieffenbachiae*, or *D. zeae* as appropriate and transductants selected on LB agar containing ampicillin. The plasmids from these transductants were extracted and analyzed by gel electrophoresis. Co-migration of the plasmid DNA samples and absence of the plasmid from the wild type (WT) controls confirmed successful plasmid transduction.

Other *Dickeya* phages with similar morphology have been described and were shown to be efficient generalized transducing phages (Matilla et al., 2014). Due to the transduction capability of certain phages shown by Matilla et al., the 67 newly-isolated phages were also tested for ability to affect horizontal gene transfer. Of these, 51 (including the 21 phages with the non-classical morphology) proved capable of transducing chromosomal markers. Twelve of the isolates, all of which had the non-classical morphology, were also tested for generalized transduction, and proved capable of transferring plasmids between *Dickeya* species. The results of transduction of the plasmid pBR322 by three of these phages are shown in Figure 2.

### 2.3. Host range

Based on the bacterial strains tested, LIMEstone1 is capable of forming plaques on strains of *D. solani* only (Adriaenssens et al., 2012b). The phages isolated during this study were tested against a variety of *Dickeya* strains, listed in Table 1, to determine their host range. The majority of the phages presented here exhibited the same host range as LIMEstone1 and were only capable of forming plaques on *D. solani* strains but not isolated representatives of other *Dickeya* species used in this study. However, eight of the phage isolates had a wider host range extending to species such as *Dickeya dieffenbachiae, Dickeya paradisiaca*, and *Dickeya zeae* (Table 2).

**Table 1:** Bacterial strains, bacteriophages, and primers used in this study.

**Bacterial strain**	**References**
*Dickeya solani* MK10	Pritchard et al., 2013a
*Dickeya dianthicola* NCPBB 453	Pritchard et al., 2013a
*Dickeya dieffenbachiae* NCPBB 2976	Pritchard et al., 2013b
*Dickeya paradisiaca* NCPBB	Pritchard et al., 2013b
*Dickeya zeae* NCPBB 3532	Pritchard et al., 2013b
*Dickeya chrysanthemi* NCPBB 402	Pritchard et al., 2013b
**Bacteriophages**	**Reference**
LIMEstone1	Adriaenssens et al., 2012b
**Primer name**	**Sequence**
oJA1	GGTTGAGGTTCATTTCTTGC
oJA2	AACGACAGGAGATTCTTYAT
oJA14	AACCACTGTTGGATTTGTCACAAGC
oJA15	AACGTCCAGTAGGGTGGAGCAT

**Table 2:** Extended host range of three groups of the isolated phages capable of infecting other species of *Dickeya*.

***Dickeya* species**	**ϕJA10, 11, and 32**	**ϕJA13, 33, and 37**	**ϕJA29 and 31**
*D. dieffenbachiae* NCPBB 2976	^+^	^+^	^+^
*D. paradisiaca* NCPBB 2511	−	−	^+^
*D. dianthicola* NCPBB 453	^+^	−	−
*D. zeae* NCPBB 3532	−	^+^	^+^
*D. chrysanthemi* NCPBB 402	^+^	−	−

+ *Denotes isolated plaque formation of the phages on the respective host*.

### 2.4. Genetic comparisons

**Figure 3:**
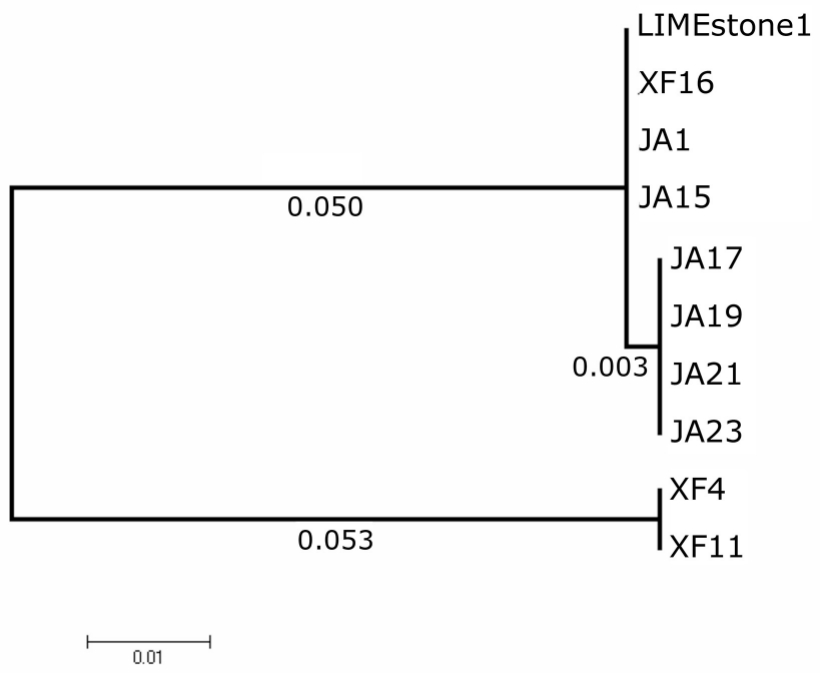
Phylogenetic tree of the DNA Polymerase (DNAP) gene from 10 phages of *Dickeya solani*. Tree was constructed using the Maximum Likelihood method with 1,134 positions in the final dataset, and the tree shown has the highest log likelihood (−2033.82).

**Figure 4:**
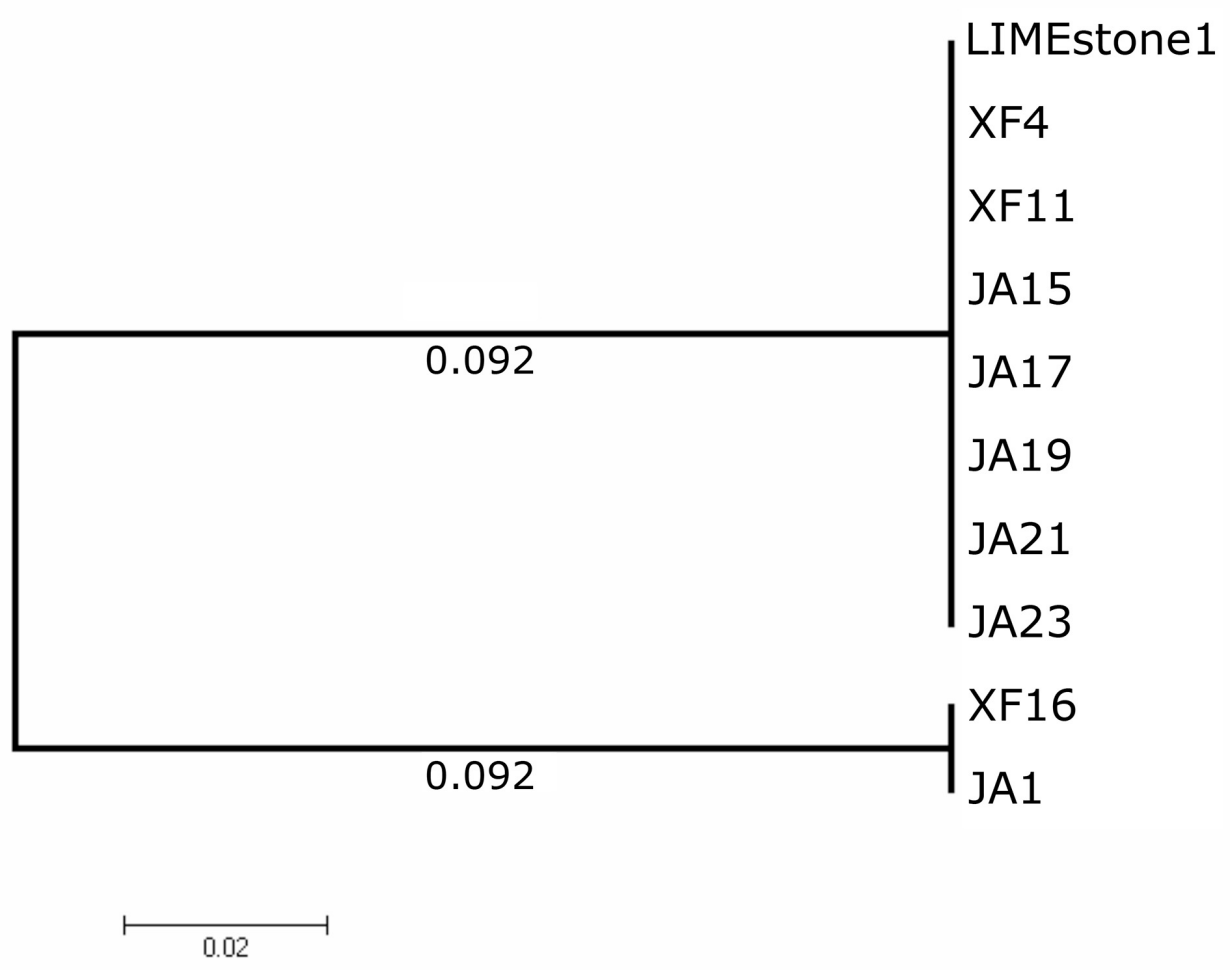
Phylogenetic tree of the Tail Spike Protein 1 (TSP1) gene from 10 phages of *Dickeya solani*. Tree was constructed using the Maximum Likelihood method with 1,593 positions in the final dataset, and the tree shown has the highest log likelihood (−3105.91). The final 20 amino acids of the TSP1 gene were trimmed from the alignment as the sequencing data for some of the phages was insufficient.

Two key genes known to be conserved between these phages, those for DNA polymerase (DNAP) and tail spike protein 1 (TSP1), were sequenced for several of the newly-isolated phages. These nucleotide sequences were then compared to those of LIMEstone1 as shown in Table 3. All of the phages in Table 3 were able to form plaques on *D. solani*. The corresponding amino acid sequences were compared between these phages and phylogenetic trees were created as shown in Figure 3 (DNAP) and Figure 4 (TSP1). These show that, in agreement with Table 3, the DNAP genes of ϕXF4 and ϕXF11 grouped together away from the other phages with a branch length of 0.053. The other phages formed two clusters that differed in a single amino acid. The TSP1 genes formed two distinct clusters, with the genes from ϕXF16 and ϕJA1 forming their own cluster with a branch length of 0.092, whereas the other phages all had identical TSP1 genes. The final 20 amino acids were trimmed from the TSP1 genes as the sequencing data for some of the phages was insufficient for tree construction.

**Table 3:** Nucleotide comparison of two conserved genes between the previously characterized LIMEstone1 and a selection of isolated phages.

	**% Identity to LIMEstone1**	
**Phage**	**DNAP**	**TSP1**
ϕXF4	90.6	100
ϕXF11	90.6	100
ϕXF16	100	83.9
ϕJA1	100	83.8
ϕJA15	99.7	100
ϕJA17	99.7	99.9
ϕJA19	99.7	100
ϕJA21	99.7	100
ϕJA23	99.7	100

### 2.5. Genomic sequencing of two new *Dickeya solani* phages

**Figure 5:**
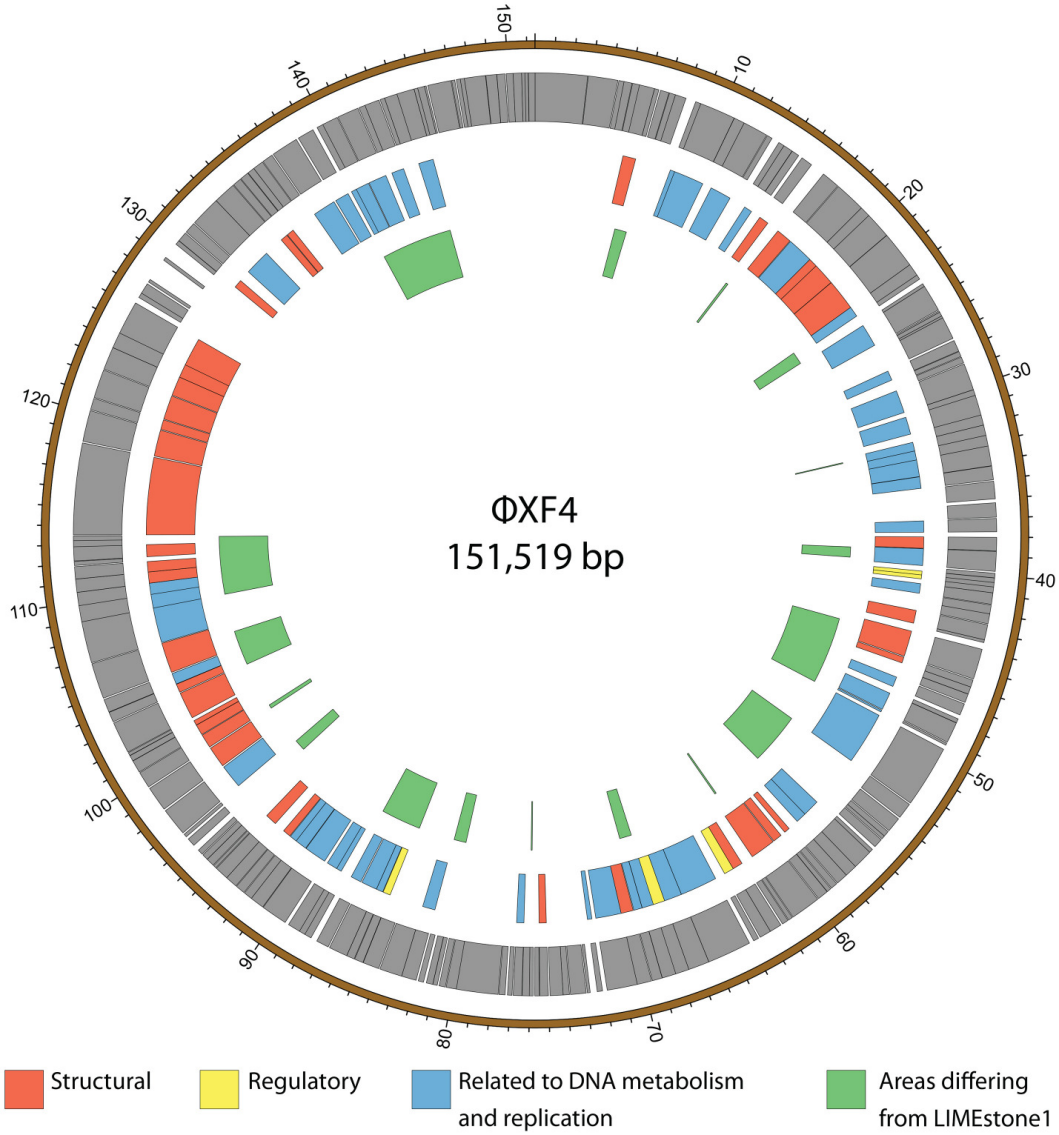
Map of the genome of ϕXF4. The outer gray ring marks open reading frames whilst the middle ring categorizes the proposed functions of these genes. The inner ring highlights the areas of the ϕXF4 genome that differ from the genome of the LIMEstone1 phage. The genome map was generated using Circos.

**Figure 6:**
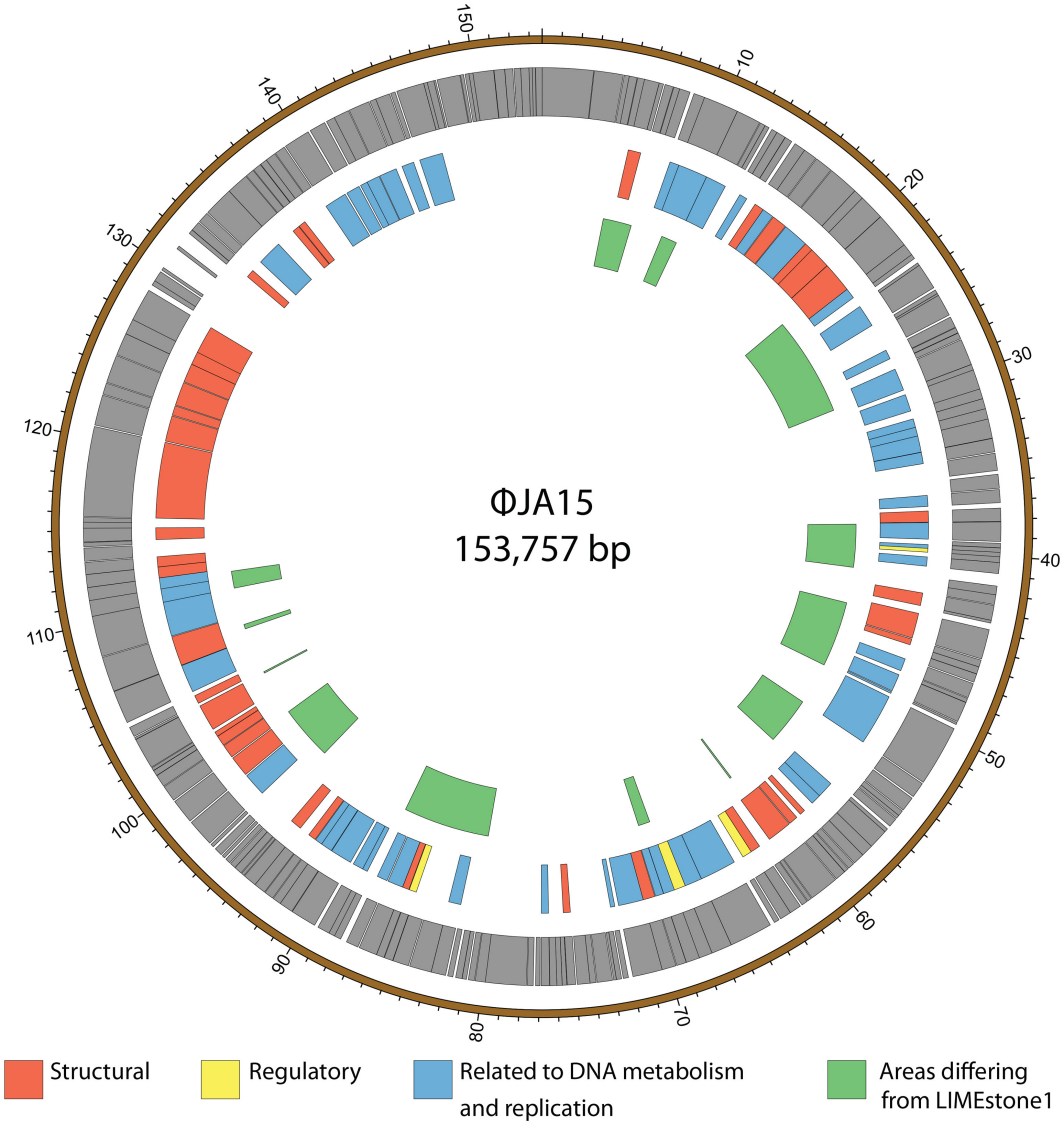
Map of the genome of ϕJA15. The outer gray ring marks open reading frames whilst the middle ring categorizes the proposed functions of these genes. The inner ring highlights the areas of the ϕJA15 genome that differ from the genome of the LIMEstone1 phage. The genome map was generated using Circos.

ϕXF4 and ϕJA15 were isolated over a year apart yet they shared the same host range and PCR amplification and preliminary sequence analysis showed 100% DNA identity in the TSP1 genes, although they differ in their DNAP genes. The full genomes of both phages were then sequenced. Both consist of circular double-stranded DNA of 151,519 and 153,757 bp, respectively. The genome of ϕXF4 has a G+C content of 49.4% and contained 185 predicted genes with lengths ranging between 116 and 4,838 nucleotides, as shown in Figure 5. ϕJA15 has a G+C content of 49.2% and contained 188 predicted genes of lengths between 122 and 4,838 nucleotides, as shown in Figure 6. Full annotation tables for the two genomes can be found in Tables S1, S2, respectively.

### 2.6. Genomic comparison

The genomes of the two new phages shared 97% DNA identity, with the main areas of difference being regions encoding endonucleases and hypothetical proteins. A comparison of both of these phages with the previously published phage LIMEstone1, showed over 95% DNA identity, with the major areas of difference consisting of genes thought to be involved in DNA replication (such as homing endonucleases and polymerases) as well as introns located throughout all three genomes. These areas of difference are highlighted in Figures 5, 6.

## 3. Discussion

The Scottish government tests all seed crops imported from outside Scotland plus 10% of Scottish-origin crops for *D. solani* and did not find any positive samples in 2016 (Scottish Government, 2016). These data are consistent with a view that *D. solani* is not yet environmentally widespread within the UK, although there have been isolated cases of *D. solani* reported in England and Wales since 2007 (Cahill et al., 2010) in crops originating from outside of the UK (Toth et al., 2016). The relative ease with which we have isolated environmental phages that infect *D. solani* therefore seems counter-intuitive given the reported paucity of the pathogen in the environment. In a restricted host range screen these phages did not form plaques on eight isolates of other Gram-negative laboratory strains such as *Pectobacterium carotovorum, Pectobacterium atrosepticum, Serratia plymuthica, Serratia marcescens, Escherichia coli*, and *Pantoea agglomerans* (data not shown). The apparently contradictory observations from phage isolations and host distribution beg the ecological question as to why *D. solani* phages are easy to find by simple enrichments. We would suggest that either *D. solani* is present in the environment around Cambridge and is not being detected, or that there is an, as yet unknown, alternative host(s) for these phages present in the environment.

All but three of the phages imaged by TEM were morphologically characterized as *Myoviridae* due to the presence of an icosahedral head and a contractile tail. Classical *Myoviridae* members, such as the coliphage T4 possess long slender tail fibers attached to the baseplate that participate in adsorption of the phage to the bacterial host. The imaged phages do not appear to have tail fibers, but instead possess shorter, clustered structures more akin to the tail spikes present in members of the *Podoviridae*. Genome analysis of the phages ϕXF4 and ϕJA15 showed genes encoding potential tail spike proteins, which show 100% sequence identity to putative tail spike protein genes in LIMEstone1. This combination of a *Myoviridae*-like morphology with tail spikes has been reported previously as a feature of the novel viral genus termed viunalikevirus (Adriaenssens et al., 2012a), named after the ViI *Salmonella* typing phage, and includes virulent phages capable of infecting a wide range of Gram-negative hosts. Members of the genus share a high degree of genome order and identity, with the major region of divergence being the genes encoding the tail spike proteins. Although, we cannot state that all phages that exhibit this morphology are definitively members of the viunalikevirus genus, we conclude it is likely. This does pose the question of whether there is some particular connection between *D. solani* and viunalikeviruses, or whether the environment around Cambridge is a particularly abundant source of this genus of phages.

A comparison of two genes from a subset of the phages isolated here, along with LIMEstone1, showed that, whilst there were some differences in DNA polymerase and TSP1 genes, these did not translate into a difference in host range and that in general these phages are highly similar. Several *D. solani* phages have now been isolated from environmental sources, including the LIMEstone phages (Adriaenssens et al., 2012b) as well as ϕD3 (Czajkowski et al., 2015) and ϕD5 (Czajkowski et al., 2014). They are all remarkably similar upon comparison of their genomes, and the two phages discussed in this study show 95% DNA identity with LIMEstone1. Much of the variation comes within the many introns and homing endonucleases found throughout the genomes of the phages, whereas structural elements are largely conserved. These introns and homing endonucleases vary in their sequence, but their position within the genomes of the phages, and thus the gene order, remains the same. It is interesting that these phages have been isolated independently in countries across Europe, and even from different environments; soil (LIMEstone1 and 2, ϕD3, and ϕD5) and water (ϕXF4 and ϕJA15), and yet they share such conservation despite their wide geographical separation.

The lytic activity of these phages, coupled with the high economic burden of *D. solani* crop phytopathogenesis, have highlighted the *D. solani* phages as potential biocontrol agents, and limited field trials have been performed (Adriaenssens et al., 2012b). Nevertheless, although these phages may have potential phage therapy features, we have shown that, 51 were capable of effecting horizontal gene transfer. We showed previously that eight candidate viunalikeviruses (including the LIMEstone phages and ϕXF4) were efficient generalized transducers of plasmid markers (Matilla et al., 2014). Consequently, we proposed that generalized transduction capacity is a characteristic trait of the viunalikevirus genus. This feature is important if these virulent phages are to be used therapeutically, as there could be potential (albeit low) for collateral transfer of bacterial virulence genes or drug resistance plasmids into unintended hosts, with unknown consequences—depending on ecological selection pressures. We therefore consider it prudent to caution against further field trials until the specific phage(s) involved are fully tested for generalized transduction capabilities.

## 4. Materials and methods

### 4.1. Bacterial strains, phages, culture media, and growth conditions

All bacterial strains used in this study are listed in Table 1. *Dickeya* species were routinely grown at 30°C in Luria broth (LB) or on LB agar plates (1.5%, wt/vol, agar). Phages were stored at 4°C in phage buffer (10 mM Tris-HCl, pH 7.4, 10 mM MgSO_4_, 0.01%, wt/vol, gelatin) over a few drops of NaHCO_3_^−^ saturated chloroform.

### 4.2. Isolation of phages

Treated sewage effluent was collected from a sewage treatment plant in Cambridge, United Kingdom (Matilla and Salmond, 2014). River water was collected from multiple sites along the River Cam. Samples were filter sterilized before 5 mL of the sample was added to 2x LB along with 500 μL of an overnight culture of *D. solani* MK10. This mixture was incubated overnight in a 250 mL flask at 30°C with shaking at 250 rpm. One milliliter of the enriched sample was mixed with 100 μL of chloroform (saturated with sodium hydrogen carbonate) and vortexed to lyse bacterial cells. The sample was centrifuged at 16,000 x g for 4 min and 10 μL of a serial dilution series of the supernatant was mixed with 200 μL of an overnight bacterial culture and 4 ml of LB top agar. This mixture was poured as an overlay on an LBA plate and incubated overnight at 30°C. Single phage plaques were picked with a sterile toothpick, placed into 100 μL phage buffer, and shaken with 40 μL of chloroform to kill any bacteria. The phages obtained were plaque purified three times. High-titre phage lysates were then obtained as described previously (Petty et al., 2006). Briefly, 10-fold serial dilutions of the phage were incubated overnight in an agar overlay as already described. Those plates exhibiting confluent lysis (seen as a mosaic-like effect in which the plaques are close to merging) were used for lysate preparation. The top agar was removed from the plate, vortexed with chloroform before sedimentation at 2,200 x g for 20 min at 4°C. The supernatant was removed and vortexed with a few drops of chloroform to produce the final lysate.

### 4.3. Transmission electron microscopy

High-titre lysates for transmission electron microscopy were obtained as described previously (Petty et al., 2006) using 0.35% (w/v) LB agarose instead of 0.35% (w/v) LB agar overlays. Ten microliters of high-titer phage lysates were adsorbed onto 400-mesh copper grids with holey carbon support films (Agar Scientific, Stansted, United Kingdom) for 30 min. The copper grids were discharged in a Quorum/Emitech K100X system (Quorum, Ringmer, United Kingdom) prior to use. After 1 min, excess phage suspension was removed with filter paper and phage samples were negatively stained by placing the grids for 5 min in 10 μL of 2% phosphotungstic acid (PTA) neutralized with sodium hydroxide, or with 10 μL of 2% uranyl acetate for 2 min. The grids were then blotted on filter paper to remove the excess solution and allowed to air dry for 10 min. Phages were examined by transmission electron microscopy in the Multi-Imaging Centre (Department of Physiology, Development and Neuroscience, University of Cambridge) using an FEI Tecnai G2 transmission electron microscope (FEI, OR, USA). The accelerating voltage was 120.0 kV, and images were captured with anAMT XR60B digital camera running Deben software.

### 4.4. Determination of host range

The host range of isolated phages was determined by plating out 10-fold serial dilutions of the phage lysates, onto agar overlays containing the six species of *Dickeya* listed in Table 1. To avoid potential confusion with “lysis from without,” only phages that produced lysis at low dilution and individual plaques were considered as being able to infect the host.

### 4.5. Transduction

To test for transduction, phage lysates were generated on donor bacterial strains carrying the desired plasmid or chromosomal marker as already described. Transduction was performed by mixing phage lysate with an overnight culture of the recipient host to achieve a multiplicity of infection of 0.1, meaning that for each phage there were 10 bacterial cells. The mixture was left on the lab bench at room temperature for 20 min, followed by incubation on a rotary wheel at 30°C for 30 min. The infected culture was then centrifuged and the bacterial pellets washed with LB twice to eliminate any remaining non-adsorbed phage. The bacterial pellets were resuspended in 1 mL LB and 100 μL aliquots were spread onto LBA plates with drug selection for the chromosomal or plasmid marker. Appropriate standard controls, which were routinely negative, were used to score for any spontaneous resistance of the recipient strain. One hundred microliters of the phage lysate was also spread onto LBA plates to confirm lysate sterility.

### 4.6. Gene amplification and analysis

Genomic DNA was extracted using Phase Lock Gel tubes (5 Prime, Hamburg, Germany) following manufacturer's instructions for isolation of Lambda DNA. DNA Polymerase (DNAP) and Tail Spike Protein 1 (TSP1) genes were amplified using Phusion polymerase (ThermoScientific, MA, USA) following standard protocols. TSP1 was amplified using the primers oJA1 and oJA2, and DNAP by the primers oJA14 and oJA15, listed in Table 1. PCR products were sequenced by GATC Biotech AG (Konstanz, Germany). Sequences were compared using NCBI Blast and the Artemis Comparison Tool (Carver et al., 2005). Phylogenetic trees were constructed using MEGA 7.0.26 (Kumar et al., 2016).

### 4.7. Genome sequencing and analysis

ϕXF4 was sequenced on the Illumina Bench Top MiSeq Sequencer (Illumina, CA, USA) at the DNA Sequencing Facility, Department of Biochemistry, University of Cambridge, UK. The resulting 138,803 reads were trimmed, quality assessed and assembled using Geneious 7.1.5 (Biomatters Ltd.), leading to higher than 100x coverage of the full genome. Gaps or single nucleotide polymorphisms were further filled or verified by Sanger sequencing to produce one contig. ϕJA15 was sequenced on the Illumina MiSeq Sequencer at MicrobesNG (Birmingham, UK). The 454,086 reads were trimmed using Trimmomatic (Bolger et al., 2014), assessed for quality using BWA-MEM (Li, 2013) and assembled using SPAdes 3.7.1 (Bankevich et al., 2012) with standard settings, leading to higher than 100x coverage of the full genome and producing one contig. Annotation of both genomes was performed using Prokka 1.11 (Seemann, 2014) using standard settings and LIMEstone1 (NC_019925.1) as a scaffold. Genome maps were generated using Circos (Krzywinski et al., 2009). Genomes were deposited in Genbank using Sequin (NCBI) and are available under accession numbers KY942057 (XF4) and KY942056 (JA15). Genomes were compared using NCBI Blast and the Artemis Comparison Tool (Carver et al., 2005).

## Author contributions

Analyzed the data, conceived and designed the experiments: AD, JA, XF, and GS. Performed the experiments: AD, JA, and XF. Wrote the paper: AD and GS.

### Conflict of interest statement

The authors declare that the research was conducted in the absence of any commercial or financial relationships that could be construed as a potential conflict of interest.
