# Supplementary material for: Environmental Bacteriophages of the Emerging Enterobacterial Phytopathogen, Dickeya solani, Show Genomic Conservation and Capacity for Horizontal Gene Transfer between Their Bacterial Hosts
Source: Front Microbiol. 2017 Aug 30;8:1654. doi: 10.3389/fmicb.2017.01654 (PMC5582154; doi:10.3389/fmicb.2017.01654)
Supplement: Supplementary file 1 [file Table1.pdf]

Supplementary Material: Environmental bacteriophages of the emerging enterobacterial phytopathogen, *Dickeya solani*, show genomic conservation and capacity for horizontal gene transfer between their bacterial hosts

Andrew Day, Jiyoon Ahn, Xinzhe Fang, and George P. C. Salmond*
*Correspondence:
Author Name: George P. C. Salmond
gpcs2@cam.ac.uk

1 SUPPLEMENTARY TABLES AND FIGURES

1.1 ϕXF4 genome annotation table

Start	End	Annotation	Group
1	2757	RIIA	
2789	4351	RIIB	
4403	4708	hypothetical protein	Hypothetical protein
4677	5102	hypothetical protein	Hypothetical protein
5133	5525	hypothetical protein	Hypothetical protein
5504	6313	unknown structural protein	Structural
6316	6549	hypothetical protein	Hypothetical protein
6638	7096	hypothetical protein	Hypothetical protein
7126	7452	hypothetical protein	Hypothetical protein
7449	8030	hypothetical protein	Hypothetical protein
8567	8857	Homing endonuclease F-LimI	Related to DNA metabolism and replication
8850	10751	DNA topoisomerase II gp39	Related to DNA metabolism and replication
10760	11332	hypothetical protein	Hypothetical protein
11329	12663	DNA topoisomerase/gyrase gp52	Related to DNA metabolism and replication
12706	12993	hypothetical protein	Hypothetical protein
13410	13724	hypothetical protein	Hypothetical protein

13724	14230	deoxycytidylate deaminase	Related to DNA metabolism and replication
14241	14648	hypothetical protein	Hypothetical protein
14860	15474	head completion protein gp4	Structural
16263	17231	tail tube associated baseplate protein gp48	Structural
17276	18919	homing endonuclease F-LimIII	Related to DNA metabolism and replication
18922	19476	baseplate wedge subunit gp53	Structural
19473	20861	baseplate hub subunit gp27	Structural
20872	22821	tail length tape measure protein	Structural
22822	23481	loader of gp41 DNA helicase gp59	Related to DNA metabolism and replication
23475	23729	hypothetical protein	Hypothetical protein
24019	24183	hypothetical protein	Hypothetical protein
24183	25607	DNA ligase gp30	Related to DNA metabolism and replication
25664	25753	hypothetical protein	Hypothetical protein
25750	25971	hypothetical protein	Hypothetical protein
25971	26087	hypothetical protein	Hypothetical protein
26084	27280	hypothetical protein	Hypothetical protein
27408	28010	homing endonuclease F-LimIV	Related to DNA metabolism and replication
28040	28312	hypothetical protein	Hypothetical protein
28353	28676	hypothetical protein	Hypothetical protein
28738	30165	DNA primase-helicase subunit gp41	Related to DNA metabolism and replication
30171	30500	hypothetical protein	Hypothetical protein
30478	31563	UvsX protein	Related to DNA metabolism and replication
31548	32090	hypothetical protein	Hypothetical protein
32090	32644	dUTP diphosphatase	Related to DNA metabolism and replication
32641	33210	dNMP kinase gp1	Related to DNA metabolism and replication

33207	34253	thymidylate synthase	Related to DNA metabolism and replication
34270	34992	thymidylate kinase	Related to DNA metabolism and replication
35067	35993	hypothetical protein	Hypothetical protein
36199	36951	hypothetical protein	Hypothetical protein
37018	37719	DNA end protector protein gp2	Related to DNA metabolism and replication
38008	38718	baseplate tail tube initiator gp54	Structural
38745	39791	ssDNA binding protein gp32	Related to DNA metabolism and replication
39890	40129	hypothetical protein	Hypothetical protein
40137	40382	late promoter transcription accessory protein gp33	Regulatory
40375	40620	regulatory protein	Regulatory
40607	40918	hypothetical protein	Hypothetical protein
40918	41520	holliday junction resolvase RuvC	Related to DNA metabolism and replication
41560	42069	hypothetical protein	Hypothetical protein
42029	42553	hypothetical protein	Hypothetical protein
42604	43410	baseplate hub subunit gp26	Structural
43410	43559	hypothetical protein	Hypothetical protein
43921	45531	baseplate hub subunit & tail lysozyme gp5	Structural
45597	45977	baseplate wedge subunit gp25	Structural
45978	46367	hypothetical protein	Hypothetical protein
46374	46835	hypothetical protein	Hypothetical protein
46918	47541	Homing endonuclease F-LimI	Related to DNA metabolism and replication
47766	47990	glutaredoxin	
48000	49106	ribonucleoside-diphosphate reductase subunit beta NrdB	Related to DNA metabolism and replication
49165	49284	Homing endonuclease F-LimI	Related to DNA metabolism and replication
49527	52829	ribonucleoside-diphosphate reductase subunit alpha NrdA	Related to DNA metabolism and replication

52913	53755	PhoH-like phosphate starvation-inducible protein	
53860	54654	endolysin	
54722	55027	hypothetical protein	Hypothetical protein
55024	55212	hypothetical protein	Hypothetical protein
55274	55762	hypothetical protein	Hypothetical protein
55958	56164	hypothetical protein	Hypothetical protein
56161	57219	DNA primase subunit gp61	Related to DNA metabolism and replication
57216	57872	homing endonuclease F-LimV	Related to DNA metabolism and replication
57872	58486	hypothetical protein	Hypothetical protein
58544	58897	unknown structural protein	Structural
58948	59187	hypothetical protein	Hypothetical protein
59197	59766	unknown structural protein	Structural
59823	61418	unknown structural protein	Structural
61463	61690	hypothetical protein	Hypothetical protein
61734	62069	hypothetical protein	Hypothetical protein
62176	62814	unknown structural protein	Structural
62814	63428	RegB protein	Regulatory
63528	63812	hypothetical protein	Hypothetical protein
64066	66402	recombination endonuclease subunit gp46	Related to DNA metabolism and replication
66405	67520	endonuclease gp47	Related to DNA metabolism and replication
67520	68251	sigma factor late transcription gp55	Regulatory
68239	68973	homing endonuclease F-LimVI	Related to DNA metabolism and replication
68982	69509	ribonuclease HI	Related to DNA metabolism and replication
69555	70322	unknown structural protein	Structural
70319	71920	ATP-dependent DNA helicase	Related to DNA metabolism and replication
72167	72445	DNA binding protein	Related to DNA metabolism and replication
72816	72968	hypothetical protein	Hypothetical protein

73027	73857	hypothetical protein	Hypothetical protein
73907	74239	hypothetical protein	Hypothetical protein
74236	74922	hypothetical protein	Hypothetical protein
75007	75456	unknown structural protein	Structural
75479	75721	hypothetical protein	Hypothetical protein
75807	76010	hypothetical protein	Hypothetical protein
76007	76381	hypothetical protein	Hypothetical protein
76394	76849	pyimidine dimer DNA glycosylase DenV	Related to DNA metabolism and replication
76910	77149	hypothetical protein	Hypothetical protein
77273	77596	acyl carrier protein	
77639	79843	vWa containing protein	
79836	80378	hypothetical protein	Hypothetical protein
80421	80753	nicotinamide phosphoribosyltransferase NadV	
80853	81086	hypothetical protein	Hypothetical protein
81093	81437	hypothetical protein	Hypothetical protein
81589	81870	hypothetical protein	Hypothetical protein
81986	82771	homing endonuclease F-LimVI	Related to DNA metabolism and replication
82773	83963	hypothetical protein	Hypothetical protein
84032	84898	hypothetical protein	Hypothetical protein
84915	85379	translational repressor protein RegA	Regulatory
85409	85831	clamp loader subunit gp62	Related to DNA metabolism and replication
85836	86825	DNA polymerase accessory protein gp44	Related to DNA metabolism and replication
86905	87573	sliding clamp gp45	Related to DNA metabolism and replication
87918	88295	hypothetical protein	Hypothetical protein
88286	88615	UvsW protein	Related to DNA metabolism and replication
88612	89280	intron I-LimI	Related to DNA metabolism and replication
89489	90880	UvsW protein	Related to DNA metabolism and replication

90909	91655	exonuclease	Related to DNA metabolism and replication
91655	92110	UvsY protein	Related to DNA metabolism and replication
92153	92653	tail completion & sheath stabilizer protein gp3	Structural
92691	93332	hypothetical protein	Hypothetical protein
93333	94061	unknown structural protein	Structural
94099	94266	hypothetical protein	Hypothetical protein
94307	94729	hypothetical protein	Hypothetical protein
94735	95055	hypothetical protein	Hypothetical protein
95115	95396	hypothetical protein	Hypothetical protein
95743	96183	hypothetical protein	Hypothetical protein
96338	96511	hypothetical protein	Hypothetical protein
96608	98083	homing endonuclease F-LimVII	Related to DNA metabolism and replication
98191	99513	major capsid protein gp23	Structural
99605	100477	scaffolding protein gp22	Structural
100523	101188	prohead protease gp21	Structural
101199	101504	prohead core protein	Structural
101515	101682	hypothetical protein	Hypothetical protein
101721	103412	portal protein	Structural
103480	104013	tail tube protein gp19	Structural
104034	104729	homing endonuclease F-LimII	Related to DNA metabolism and replication
104813	106711	tail sheath protein gp18	Structural
106764	108971	terminase large subunit gp17	Related to DNA metabolism and replication
108961	109839	homing endonuclease F-LimIX	Related to DNA metabolism and replication
109817	110518	terminase small subunit gp16	Related to DNA metabolism and replication
110521	111216	tail sheath stabilizer gp15	Structural
111219	111869	neck protein gp14	Structural
111929	112141	hypothetical protein	Hypothetical protein
112169	112921	neck protein gp13	Structural
112911	113249	hypothetical protein	Hypothetical protein

113230	113481	hypothetical protein	Hypothetical protein
113532	118370	unknown structural protein	Structural
118454	120100	tailspike protein	Structural
120155	120769	tailspike protein	Structural
120820	122334	tailspike protein	Structural
122387	123598	unknown structural protein	Structural
123601	124455	baseplate wedge subunit gp7	Structural
124439	126217	baseplate wedge subunit gp6	Structural
126550	126825	hypothetical protein	Hypothetical protein
126815	127387	hypothetical protein	Hypothetical protein
127502	127660	hypothetical protein	Hypothetical protein
128891	129073	hypothetical protein	Hypothetical protein
129941	130177	hypothetical protein	Hypothetical protein
130186	130704	unknown structural protein	Structural
130775	131362	hypothetical protein	Hypothetical protein
131432	133039	homing endonuclease F-LimX	Related to DNA metabolism and replication
133041	134231	threonine ammonia lyase	
134271	134729	unknown structural protein	Structural
134765	135223	unknown structural protein	Structural
135249	135623	hypothetical protein	Hypothetical protein
135625	136185	hypothetical protein	Hypothetical protein
136227	136799	hypothetical protein	Hypothetical protein
136879	138312	DNA polymerase gp43	Related to DNA metabolism and replication
138441	139319	intron I-LimII	Related to DNA metabolism and replication
139561	139911	DNA polymerase gp43	Related to DNA metabolism and replication
139898	140740	intron I-LimIII	Related to DNA metabolism and replication
140787	141920	DNA polymerase gp43	Related to DNA metabolism and replication
141982	142323	hypothetical protein	Hypothetical protein
142320	143096	5'(3') deoxyribonucleotidase	Related to DNA metabolism and replication

143163	143411	thioredoxin	
143411	144097	hypothetical protein	Hypothetical protein
144090	145019	homing endonuclease F-LimXI	Related to DNA metabolism and replication
145022	145219	hypothetical protein	Hypothetical protein
145216	145596	hypothetical protein	Hypothetical protein
145577	145675	hypothetical protein	Hypothetical protein
145759	146994	hypothetical protein	Hypothetical protein
147031	147198	hypothetical protein	Hypothetical protein
147280	147492	hypothetical protein	Hypothetical protein
147489	147707	hypothetical protein	Hypothetical protein
147704	148819	hypothetical protein	Hypothetical protein
148839	149408	hypothetical protein	Hypothetical protein
149413	149841	hypothetical protein	Hypothetical protein
149904	150269	hypothetical protein	Hypothetical protein
150266	150712	hypothetical protein	Hypothetical protein
150709	150909	hypothetical protein	Hypothetical protein
150881	151054	hypothetical protein	Hypothetical protein
151065	151412	hypothetical protein	Hypothetical protein

Table S1: Annotation table for ϕXF4. Annotations performed using Prokka 1.11 using LIMEstone1 (NC_019925.1) as a scaffold. Numbers represent base pairs from the start of the contig and are therefore an arbritary start point. Genes were manually sorted as noted in the 'Group' column and these groups were used in Fig 3.

1.2 ϕJA15 genome annotation table

**Start**	**End**	**Annotation**	**Group**
1	2757	Phage rIIA lysis inhibitor	
2789	4351	Phage lysis inhibitor # T4-like rIIA-rIIB membrane associated #T4 GC1698	
4403	4708	hypothetical protein	Hypothetical protein
4677	5102	hypothetical protein	Hypothetical protein
5292	5525	hypothetical protein	Hypothetical protein
5504	6313	Phage tail fiber	Structural
6316	6549	hypothetical protein	Hypothetical protein
6638	7096	putative histone-like protein	
7126	7452	hypothetical protein	Hypothetical protein
7895	7743	hypothetical protein	Hypothetical protein
8084	8857	Phage-associated homing endonuclease	Related to DNA metabolism and replication

8850	10748	DNA topoisomerase, phage-associated	Related to DNA metabolism and replication
10750	12081	DNA topoisomerase, phage-associated	Related to DNA metabolism and replication
12124	12411	hypothetical membrane protein	Hypothetical protein
12828	13142	hypothetical protein	Hypothetical protein
13142	13648	dCMP deaminase (EC 3.5.4.12)	Related to DNA metabolism and replication
13659	14066	Membrane-flanked domain	Hypothetical protein
14892	14278	Phage head completion protein	Structural
15629	14892	Phage-associated homing endonuclease	Related to DNA metabolism and replication
15680	16648	gp48 baseplate tail tube cap	Structural
16693	18336	Phage protein	Hypothetical protein
18339	18893	baseplate wedge component gp53	Structural
18890	20278	hypothetical protein	Hypothetical protein
20289	22238	hypothetical protein	Hypothetical protein
22904	22239	Phage DNA helicase loader	Related to DNA metabolism and replication
23152	22898	hypothetical protein	Hypothetical protein
23465	23154	hypothetical membrane protein	Hypothetical protein
23595	23440	hypothetical protein	Hypothetical protein
25019	23595	DNA ligase, phage-associated	Related to DNA metabolism and replication
25338	25529	hypothetical protein	Hypothetical protein
26689	25496	Phage protein	Hypothetical protein
27745	27449	hypothetical protein	Hypothetical protein
28086	27763	hypothetical protein	Hypothetical protein
29575	28148	DNA primase/helicase, phage-associated	Related to DNA metabolism and replication
29910	29581	hypothetical protein	Hypothetical protein
30973	29888	Phage recombination protein	
31500	30958	hypothetical protein	Hypothetical protein
32054	31500	putative dUTP diphosphatase	Related to DNA metabolism and replication

32620	32051	putative deoxynucleotide monophosphate kinase	Related to DNA metabolism and replication
33663	32617	Thymidylate synthase (EC 2.1.1.45)	
34402	33680	hypothetical protein	Hypothetical protein
35403	34477	hypothetical protein	Hypothetical protein
36361	35609	hypothetical protein	Hypothetical protein
37129	36428	Phage DNA end protector during packaging	Related to DNA metabolism and replication
37184	38128	gp19 tail tube monomer	Structural
39201	38155	Single stranded DNA-binding protein, phage-associated	Related to DNA metabolism and replication
39539	39300	hypothetical protein	Hypothetical protein
39792	39547	Gp33 T4-like late promoter transcription accessory protein	Structural
40030	39785	hypothetical protein	Hypothetical protein
40328	40017	hypothetical membrane protein	Hypothetical protein
40876	40328	hypothetical protein	Hypothetical protein
42080	41589	hypothetical protein	Hypothetical protein
42786	43439	Gp26 baseplate hub subunit	Structural
43439	43588	hypothetical protein	Hypothetical protein
43950	45560	Phage baseplate hub subunit (T4-like gp5) / Phage tail lysozyme (T4-like gp5)	Structural
45566	46006	putative Gp25 baseplate wedge subunit	Structural
46183	46007	hypothetical protein	Hypothetical protein
46864	46403	hypothetical protein	Hypothetical protein
47747	46947	Phage-associated homing endonuclease	Related to DNA metabolism and replication
48019	47795	putative glutaredoxin	
49135	48029	Ribonucleotide reductase of class Ia (aerobic), beta subunit (EC 1.17.4.1)	Related to DNA metabolism and replication
52858	49556	Ribonucleotide reductase of class Ia (aerobic), alpha subunit (EC 1.17.4.1)	Related to DNA metabolism and replication
53784	52942	Phosphate starvation-inducible protein PhoH, predicted ATPase	
54683	53889	Putative phage-encoded peptidoglycan binding protein	
55056	54751	hypothetical protein	Hypothetical protein

55241	55053	hypothetical protein	Hypothetical protein
55791	55303	hypothetical protein	Hypothetical protein
55858	55980	hypothetical protein	Hypothetical protein
56193	55987	hypothetical protein	Hypothetical protein
57047	56190	Phage-associated DNA primase (EC 2.7.7.-)	Related to DNA metabolism and replication
57901	57245	Phage-associated homing endonuclease	Related to DNA metabolism and replication
58515	57901	Phage protein	Hypothetical protein
58926	58573	Phage protein	Hypothetical protein
59216	58977	hypothetical protein	Hypothetical protein
59795	59226	hypothetical protein	Hypothetical protein
61447	59852	hypothetical protein	Hypothetical protein
61719	61492	hypothetical protein	Hypothetical protein
62098	61763	hypothetical protein	Hypothetical protein
62843	62205	hypothetical protein	Hypothetical protein
63232	62843	Vs.1 conserved hypothetical protein	
63841	63557	hypothetical protein	Hypothetical protein
66431	64095	Phage recombination-related endonuclease Gp46	
67549	66434	Phage recombination-related endonuclease Gp47	
68280	67549	Gp55 T4-like sigma factor involved in late transcri-ption	Regulatory
69002	68268	Protein of Unknown Function	Hypothetical protein
69538	69011	Ribonuclease HI (EC 3.1.26.4)	Related to DNA metabolism and replication
69584	70351	hypothetical protein	Hypothetical protein
72066	70348	DNA helicase, phage-associated	Related to DNA metabolism and replication
72474	72196	DNA-binding protein HU-beta	Related to DNA metabolism and replication
72717	72565	hypothetical protein	Hypothetical protein
72997	72845	hypothetical protein	Hypothetical protein
73886	73056	hypothetical protein	Hypothetical protein
74265	73936	hypothetical protein	Hypothetical protein
74951	74265	hypothetical protein	Hypothetical protein
75485	75036	hypothetical protein	Hypothetical protein
76040	75753	hypothetical protein	Hypothetical protein
76411	76037	hypothetical protein	Hypothetical protein

76879	76424	Endonuclease V (EC 3.1.25.1)	Related to DNA metabolism and replication
77179	76940	hypothetical protein	Hypothetical protein
77626	77303	hypothetical protein	Hypothetical protein
79873	77669	Phage protein	Hypothetical protein
80408	79866	hypothetical protein	Hypothetical protein
80783	80451	Nicotinamide phosphoribosyltransferase (EC 2.4.2.12)	
81116	80883	hypothetical protein	Hypothetical protein
81467	81123	hypothetical protein	Hypothetical protein
81900	81619	Phage protein	Hypothetical protein
82801	82016	Protein of Unknown Function	Hypothetical protein
83993	82803	hypothetical protein	Hypothetical protein
84928	84062	hypothetical protein	Hypothetical protein
85409	84945	Phage endoribonulcease translational repressor of early genes, regA	Regulatory
85861	85439	Phage DNA polymerase clamp loader subunit Gp62	Related to DNA metabolism and replication
86909	85866	Replication factor C small subunit / Phage DNA polymerase clamp loader subunit	Related to DNA metabolism and replication
87603	86935	Sliding clamp DNA polymerase accessory protein, phage associated	Related to DNA metabolism and replication
87948	88325	hypothetical protein	Hypothetical protein
88645	88316	DNA helicase UvsW	Related to DNA metabolism and replication
89541	88642	hypothetical protein	Hypothetical protein
90910	89519	DNA helicase, phage-associated	Related to DNA metabolism and replication
91685	90939	hypothetical protein	Hypothetical protein
92140	91685	putative DNA repair/recombination protein UvsY	Related to DNA metabolism and replication
92683	92183	Phage tail completion protein	Structural
92712	93362	hypothetical protein	Hypothetical protein
94091	93363	Phage protein	Hypothetical protein
94296	94129	hypothetical protein	Hypothetical protein
94759	94337	hypothetical protein	Hypothetical protein

95085	94765	hypothetical protein	Hypothetical protein
95426	95145	hypothetical protein	Hypothetical protein
95690	95529	hypothetical protein	Hypothetical protein
96213	95773	hypothetical protein	Hypothetical protein
98113	96638	Phage protein	Hypothetical protein
99540	98218	Gp23 major head protein	Structural
100504	99632	Phage prohead assembly (scaffolding) protein	Structural
101218	100550	Phage prohead core scaffold protein and protease #T4-like phage gp21 #T4 GC0129	Structural
101531	101226	hypothetical protein	Hypothetical protein
101709	101542	hypothetical protein	Hypothetical protein
103439	101748	Gp20 portal vertex protein of head	Structural
104137	103634	Phage tail tube monomer #T4-like phage Gp19	Structural
104458	106248	Phage protein	Hypothetical protein
108173	106275	gp18 tail sheath protein	Structural
110433	108226	Gp17 terminase DNA packaging enzyme large subunit	Related to DNA metabolism and replication
111310	110423	Protein of Unknown Function	Hypothetical protein
111980	111279	Gp16 terminase DNA packaging enzyme small subunit	Related to DNA metabolism and replication
112678	111983	Gp15 proximal tail sheath stabilization protein	Structural
113331	112681	Phage neck protein # Gp14	Structural
114166	114390	hypothetical protein	Hypothetical protein
115171	114419	Gp13 neck protein	Structural
115499	115161	hypothetical protein	Hypothetical protein
115731	115480	hypothetical protein	Hypothetical protein
120620	115782	Phage virulence-associated VriC protein	
122350	120704	Phage tail fibers	Structural
124584	123070	hypothetical protein	Hypothetical protein
125848	124637	hypothetical protein	Hypothetical protein
126705	125851	hypothetical protein	Hypothetical protein
128404	126689	gp6 baseplate wedge subunit	Structural
131140	131322	hypothetical protein	Hypothetical protein
132098	131958	hypothetical protein	Hypothetical protein
132190	132426	hypothetical protein	Hypothetical protein
132435	132953	Phage protein	Hypothetical protein
133024	133611	hypothetical protein	Hypothetical protein
133681	135288	Phage protein	Hypothetical protein
135290	136480	hypothetical protein	Hypothetical protein
136520	136978	hypothetical protein	Hypothetical protein
137014	137472	hypothetical protein	Hypothetical protein

137872	137498	hypothetical protein	Hypothetical protein
138383	137874	hypothetical protein	Hypothetical protein
139048	138476	hypothetical protein	Hypothetical protein
139128	140561	DNA polymerase (EC 2.7.7.7), phage-associated	Related to DNA metabolism and replication
140690	141568	hypothetical protein	Hypothetical protein
141682	142161	DNA polymerase (EC 2.7.7.7), phage-associated	Related to DNA metabolism and replication
142990	142148	Phage-associated homing endonuclease	Related to DNA metabolism and replication
143037	144170	DNA polymerase (EC 2.7.7.7), phage-associated	Related to DNA metabolism and replication
144233	144574	hypothetical protein	Hypothetical protein
144571	145350	hypothetical protein	Hypothetical protein
145360	145665	hypothetical protein	Hypothetical protein
145662	147257	Phage-associated homing endonuclease	Related to DNA metabolism and replication
147260	147457	hypothetical membrane protein	Hypothetical protein
147454	147834	hypothetical protein	Hypothetical protein
147997	149232	hypothetical protein	Hypothetical protein
149269	149436	hypothetical membrane protein	Hypothetical protein
149557	149730	hypothetical protein	Hypothetical protein
149793	149945	hypothetical protein	Hypothetical protein
150253	149897	hypothetical protein	Hypothetical protein
150284	151057	hypothetical protein	Hypothetical protein
151077	151646	hypothetical protein	Hypothetical protein
151651	152079	hypothetical protein	Hypothetical protein
152130	152507	hypothetical protein	Hypothetical protein
152504	152950	hypothetical protein	Hypothetical protein
152947	153147	hypothetical protein	Hypothetical protein
153303	153650	hypothetical protein	Hypothetical protein

Table S2: Annotation table for ϕJA15. Annotations performed using Prokka 1.11 using LIMEstone1 (NC_019925.1) as a scaffold. Numbers represent base pairs from the start of the contig and are therefore an arbitrary start point. Genes were manually sorted as noted in the 'Group' column and these groups were used in Fig 4.
